# Cell cycle checkpoint activity in the malaria parasite *Plasmodium falciparum*

**DOI:** 10.1128/msphere.00341-26

**Published:** 2026-07-10

**Authors:** Monique K. Johnson, Juliana Naldoni, William H. Lewis, Ross F. Waller, Catherine J. Merrick

**Affiliations:** 1 Department of Pathology, University of Cambridge 2152, Cambridge, United Kingdom; 2 Department of Biochemistry, University of Cambridge 2152, Cambridge, United Kingdom; Institut Pasteur de Montevideo, Montevideo, Montevideo, Uruguay

**Keywords:** Malaria, *Plasmodium*, cell cycle, checkpoint, PI3K

## Abstract

*Plasmodium* spp. have different modes of cell division from most eukaryotes. Little is known about how these are controlled, and cell cycle checkpoints are particularly poorly characterized. However, parasites can arrest their cell cycle when treated with the frontline antimalarial drug artemisinin, and artemisinin-resistant parasites can modulate their cell cycle progression, so it is important to understand these aspects of *Plasmodium* biology. Here, we show that *P. falciparum* displays hallmarks of an intra-S-phase checkpoint when exposed to DNA damage, including acute reduction of DNA replication and phosphorylation of a putative damage-marker histone. Compounds that inhibit human checkpoint kinases can inhibit this arrest of DNA replication and synergize with DNA damage in parasite killing. This suggests the existence of checkpoint kinase activity in *P. falciparum*, yet these kinases have no clear homologues in *Plasmodium* genomes. Their closest homologs are the phosphatidylinositol lipid kinases. We hypothesize that phosphatidylinositol 3-kinase—which is reportedly upregulated in artemisinin-resistant parasites—may moonlight in this role, and we characterize this essential kinase for the first time via expansion microscopy. Finally, we show that the cryptic checkpoint-kinase activity may also regulate the ring-stage survival phenotype after artemisinin damage, which resembles a G1/S checkpoint. Hence, we suggest that checkpoint kinase inhibitors are candidates for synergy with artemisinin.

**IMPORTANCE:**

Malaria parasites infect red blood cells, wherein they replicate to produce many new parasites. This is unusual because most cells replicate simply by copying their genome and splitting in half (called binary fission), but malaria parasites make ~20 genome copies and then partition them simultaneously into 20 new cells (called schizogony). Here, we studied how schizogony is controlled: in particular, are there “checkpoints,” i.e., pathways that can pause the cell cycle? We found that DNA damage did cause checkpoint hallmarks, yet the key proteins that enforce this in other cells are absent in malaria parasites. Furthermore, this checkpoint activity may be involved in the response to an antimalarial drug, in which parasites pause their cycle before active replication begins. This implies that inhibiting the checkpoint could exacerbate parasite killing by such drugs. Cancer therapies often work like this—by damaging DNA and also preventing the cancer cells from repairing it.

## INTRODUCTION

*Plasmodium falciparum* is the most important cause of human malaria, giving rise to over half a million deaths and hundreds of millions of clinical cases per year (1). Besides being a major human pathogen, it is also a highly unusual protozoan organism, distantly related to humans and other model organisms, with distinctive features of cell and molecular biology. One of these is a non-canonical cell cycle. Instead of dividing by binary fission, as in most model organisms, *Plasmodium* parasites divide primarily by schizogony. After invading a host erythrocyte, they execute multiple, asynchronous rounds of genome replication and nuclear division, generating multiple nuclei within the same cytoplasm. This is followed by a relatively synchronous mass cytokinesis event, partitioning ~20 nuclei and organelles into individual merozoites.

The complex events of schizogony are now being dissected in molecular and cellular detail (2–5), but fundamental questions remain about how the process is regulated. Schizogony is not easily mapped onto the cell cycle phases of G1, S, G2, and M. After invading a host cell, the parasite undergoes a pre-replicative growth phase broadly analogous to G1 (6). The haploid genome is then repeatedly replicated in a continuous phase of repeated S and M phases, without once-and-only-once regulation, generating many new haploid nuclei. Different nuclei cease to replicate at different times and divide at different times, so there is no well-defined, single G2 and M-phase (3).

*Plasmodium* replication is not controlled by conventional cyclin-dependent kinase activity (7), nor is it apparently governed by conventional cell cycle checkpoints, which usually operate through specific checkpoint kinases to halt cell cycle progression at the G1/S transition, within S-phase, at G2/M and at spindle-assembly/mitosis. Respectively, these checkpoints delay the start of S-phase, arrest replication if DNA damage or replication stress is detected, and prevent mitosis if a genome is incompletely replicated (8). The *Plasmodium* genus, however, encodes no clear homologs of the checkpoint kinases conserved in most eukaryotes (animals, fungi, and plants), e.g., *S. cerevisiae* Mec1 and Rad53 or human ATM/ATR and Chk1/Chk2 (7). (By contrast, some parasites in the apicomplexan phylum, like *Toxoplasma*, do retain homologs of the “master regulators“ Mec1/ATM/ATR, which are phosphoinositide-3-kinase-like kinases (PIKKs), and checkpoints are better characterized in *Toxoplasma* [9–11]).

Nevertheless, it seems unlikely that *Plasmodium* is entirely without checkpoints. Eukaryotic cells (not only in metazoans, which must guard against cancer, but also in protozoans) generally do pause their cell cycles to ensure effective DNA repair. *Plasmodium* encodes most of the known DNA repair pathways (7, 12) and generates relatively low rates of SNPs, indels, and translocations during normal replication (13).

There is some evidence for a G1/S-like checkpoint: pre-replicative rings can undergo “temporary growth arrest (TGA)” (14) in response to nutritional (15), temperature (16), or antimalarial-drug (17, 18) stresses. TGA is particularly important under stress caused by the antimalarial drug artemisinin because it can contribute to treatment failure. However, there is no molecular evidence as yet that conventional checkpoint effectors are involved. A G1/S checkpoint usually involves the cyclin/CDK complexes that drive S-phase entry, influenced positively by growth factors or negatively by damage-responsive kinases like PIKKs. TGA in *Plasmodium*, by contrast, is defined only as a “non-specific stress-response survival mechanism” (14): induced by multiple stresses, possibly via multiple molecular pathways.

An intra-S-phase checkpoint in a mammalian or yeast cell usually slows the pace of S-phase specifically after DNA damage or replication stress (19, 20). In *Plasmodium*, the transcriptional cascade that drives the cell cycle can slow down after DNA damage, with DNA repair proteins and chromatin modifications being upregulated (21), but the signals and transducers for any potential checkpoint are unknown. *Plasmodium* generally lacks validated markers for DNA damage and for direct responses to it. Indeed, the multi-nucleate, asynchronous nature of schizogony raises questions about how an intra-S-phase checkpoint should or could be enforced. Replication itself is apparently regulated per-nucleus, probably via individual accumulations of PCNA, together with non-canonical, chromatin-bound CDK-related kinases like CRK4 (22). Nevertheless, diffusible checkpoint kinases, potentially affecting the whole cell, may not be incompatible with schizogony—at least in response to exogenous DNA damage.

Finally, a G2/M checkpoint may be entirely absent because some *P. falciparum* mutants perform grossly aberrant genome segregation but still undergo cytokinesis (23). (One of the parasite’s non-cycling cyclins, PfCyc1, has actually been reported to have a role at cytokinesis [24].) In fact, even in non-mutants, certain phases of the lifecycle produce a high rate of zoids—this occurs particularly during gametogenesis, wherein cytokinesis proceeds even if genome replication fails entirely (25, 26).

Here, we set out to investigate checkpoint responses to DNA damage in *Plasmodium*, using newly developed molecular and cellular tools. We show that the hallmarks of an S-phase checkpoint do occur in *P. falciparum* despite the apparent absence of canonical PIKKs.

## RESULTS

### Evidence for DNA-damage-responsive checkpoint activity in *P. falciparum*

We used two complementary tools to investigate the response to DNA damage during S-phase in *P. falciparum*, focusing at the levels of DNA and protein. First, we labeled nascent DNA in replicating parasites with a quantifiable modified nucleotide. This tested whether DNA replication would acutely arrest after damage: this is a hallmark of the intra-S-phase checkpoint (19, 20), but it can occur in several ways, including physical replication fork stalling, checkpoint-mediated slowing of fork movement, and checkpoint-mediated inhibition of origin firing. Second, therefore, we measured phosphorylation of histone H2A, which was recently identified as being analogous to histone H2AX phosphorylation in mammalian cells (27). Canonically, H2AX is phosphorylated at chromatin sites of DNA damage (28, 29). H2AX is absent in *Plasmodium*, but the non-variant histone H2A can nevertheless be phosphorylated (27). Importantly, this indicates an in *trans*, kinase-driven response that is genuinely “checkpoint-like.”

To induce DNA damage, we used the well-characterized alkylating agent methyl methane sulphonate (MMS), which alkylates DNA bases, particularly guanine and adenine (30). In mammalian cells, it induces an intra-S-phase checkpoint (19). In *P. falciparum* parasites, it leads to DNA breakage, as measured by comet assays (31) and concomitantly delays the cell cycle (21), but its effect as a checkpoint inducer has not been explicitly addressed. In the *P. falciparum* 3D7 strain, MMS had a 48h IC_50_ of ~96 µM (see Fig. S1A at https://doi.org/10.5281/zenodo.21245733). Having established this, we performed highly acute assays over just 1–2 h, using 10× or 20× IC_50_, to minimize non-specific or secondary effects. We confirmed that parasites could substantially recover from this level of MMS exposure within one cell cycle, although their maturation was delayed, as reported previously (21), and there was also a reduction in merozoite numbers in mature schizonts (see Fig. S2 at https://doi.org/10.5281/zenodo.21245733).

**Fig 1:**
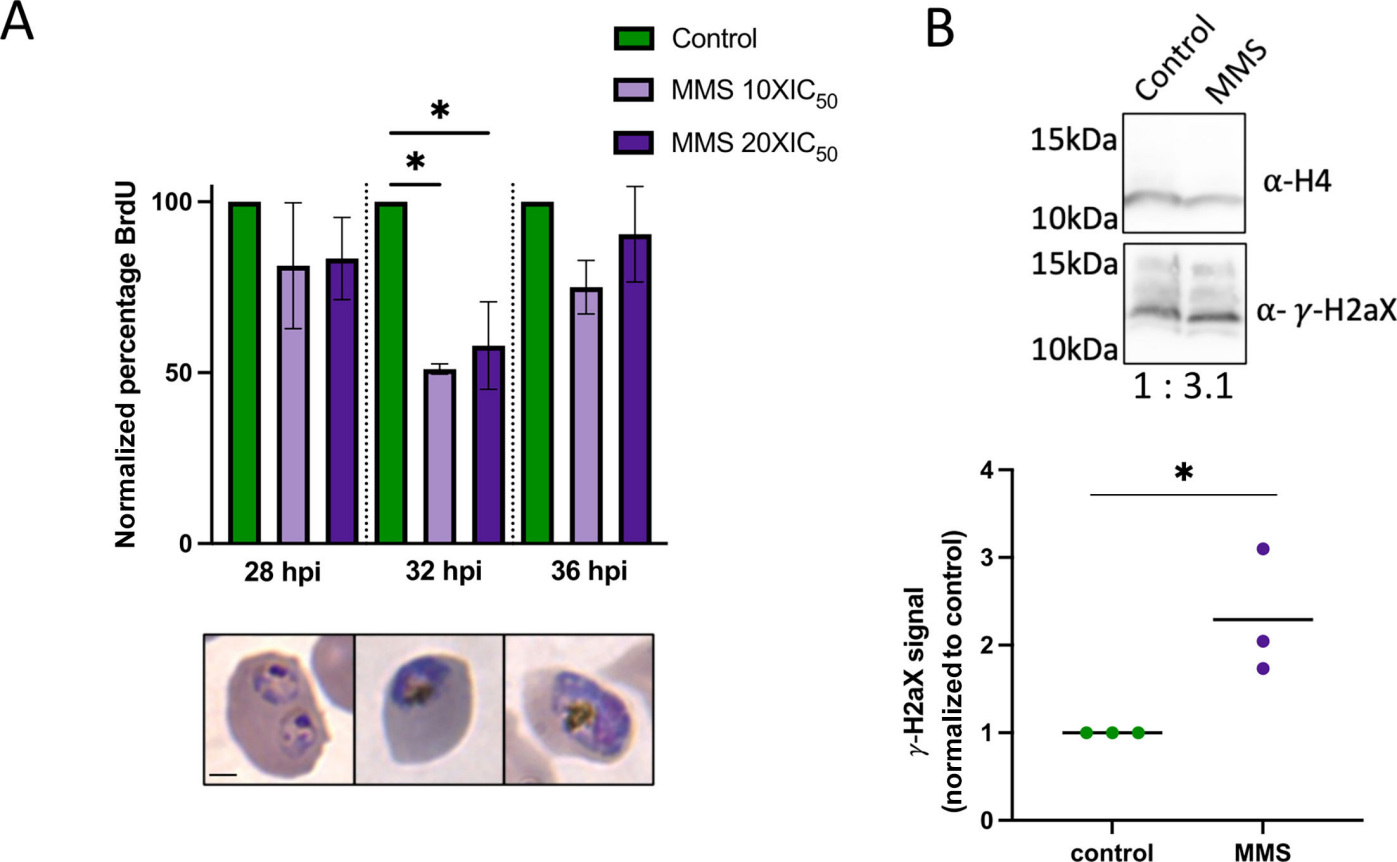
Evidence for DNA-damage-responsive checkpoint activity in *P. falciparum.* (**A**) BrdU incorporation into populations of trophozoites at three stages, after treatment with MMS, as measured by ELISA. Parasites were treated with MMS for 1 h, and BrdU was added for the latter 30 min. Means of two biological replicates, conducted in technical triplicate, are shown; error bars show range. MMS treatments: 10× IC_50_, 960 µM; 20× IC_50_, 1.92 mM. For each parasite stage, statistical testing was via ordinary one-way ANOVA and post hoc Tukey’s multiple comparisons test. *, *P*-value < 0.05; comparisons not shown, ns. Pictures show examples of trophozoites at each timepoint. Scale bar, 2 µm. (**B**) Representative western blot of phosphorylated histone in trophozoite parasite lysate treated with MMS (2 h, 10× IC_50_). Phosphorylated histone signal was quantified vs control histone H4: relative quantification is shown below the blot. Graph shows quantifications from three biological replicate blots, relative to either H4 or Hsp70 as a control protein. Unpaired two-tailed *t*-test: *P*-value = 0.0354.

DNA replication was acutely reduced in 30 min following a 30-min exposure to MMS at 10× or 20× IC_50_. Some reduction occurred throughout the entire S/M period (~28–36 hours post invasion, hpi), but it was most pronounced when DNA was damaged in early trophozoites at ~32 hpi (Fig. 1A). Within a similar timeframe, histone H2A phosphorylation was moderately elevated (Fig. 1B). We recently reported that several DNA damaging agents, including MMS, could cause H2A phosphorylation (32), but that the upregulation in trophozoite stages was modest—usually less than twofold over background levels—making it difficult to measure consistently. This was equally true when MMS was used to induce H2A phosphorylation here (Fig. 1B). (Previously published work measured H2A phosphorylation in pre-replicative rings exposed to ionizing radiation (27), whereas we measured it in actively replicating trophozoites, wherein the background signal may be higher.) Therefore, we chose the replication-reduction assay as a more robust readout for checkpoint activity in subsequent experiments.

### Absence of clear checkpoint mediators in *P. falciparum*

**Fig 2:**
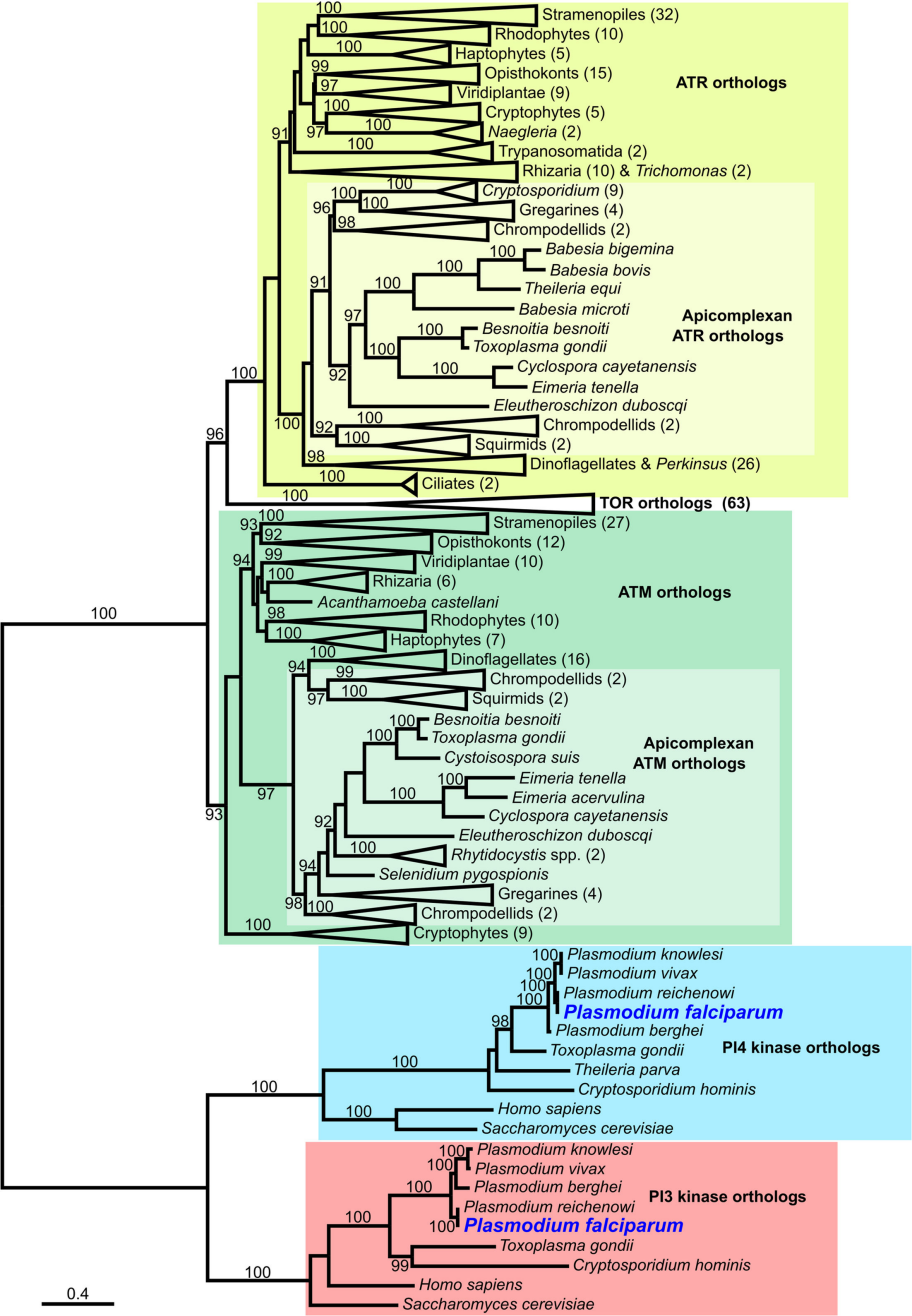
*Plasmodium* encodes no homologs of PIKK checkpoint kinases. A phylogeny inferred for amino acid protein sequences of ATR, ATM, and TOR homologs sampled from broad eukaryote groups. Apicomplexa homologs of PI3K and PI4K (the most closely related proteins to ATR and ATM present in *Plasmodium*) and characterized homologs of PI3K and PI4K from *Saccharomyces cerevisiae* and *Homo sapiens* were included as an outgroup. Support values, displayed as percentages, were generated from 1,000 ultrafast bootstrap replicates (75) and only support values ≥90, indicating strongly supported clades, are shown. The scale bar represents the number of amino acid substitutions per site. A fully expanded version of this tree with no collapsed clades and displaying sequence accessions is provided in the source data at https://doi.org/10.5281/zenodo.21245733.

When the intra-S-phase checkpoint is triggered in other eukaryotes, both histone phosphorylation and arrested DNA replication are transduced through PIKKs (20, 28). Since Fig. 1 shows that these phenotypes also appear in *P. falciparum,* a transducing kinase should be present. However, orthologs of ATM/ATR are not found in the *P. falciparum* genome (7). To test for the presence or loss of such orthologs more thoroughly, we performed an extensive phylogenetic analysis of PIKK homologs throughout apicomplexans and other major eukaryotic lineages (Fig. 2). In this analysis, the lipid kinases PI3K and PI4K formed an outgroup relative to all ATM/ATR-like proteins. In eukaryotes, PI3/4Ks are generally the closest relatives of PIKKs (33), and we did not seek to re-establish this (e.g., by surveying apicomplexan genomes for any other kinases resembling PIKKs); we simply sought to establish patterns of PIKK presence/loss in apicomplexans.

ATR homologs were found in most apicomplexans, and some—like *T. gondii*—clearly retained homologs of both ATM and ATR. Other apicomplexans, including *Cryptosporidium*, *Babesia,* and *Theileria*, had independently lost the ATM homolog, indicating a propensity for loss of PIKKs in Apicomplexa. *Plasmodium* spp., however, had lost both ATM and ATR, retaining no apparent PIKKs at all. This raised the conundrum of what the checkpoint-transducing kinase could be: if the whole checkpoint pathway has radically diverged in *Plasmodium,* then it could be an entirely different, non-PIKK-like entity. However, we hypothesized that a related protein might “moonlight” in this role. The closest PIKK homologs in any *Plasmodium* genome were the lipid kinases PI3K and PI4K (Fig. 2; see Fig. S3 at https://doi.org/10.5281/zenodo.21245733), which were also present throughout apicomplexans (34). (*Theileria*, unusually, was found to have lost its PI3K homolog, possibly due to a lifecycle conducted free in the cytoplasm of leukocytes, which may supply this activity.).

Notably, the closest *Plasmodium* PIKK homolog, PI3K, is reportedly overexpressed in artemisinin-resistant parasites (35), which can alter their cell cycle dynamics. Furthermore, a recent analysis of the steady-state locations of mature schizont-stage *P. falciparum* proteins by spatial proteomics assigned PI3K as a nucleus-located protein, whereas PI4K was assigned to cytoplasmic transport vesicles (see Fig. S4A https://doi.org/10.5281/zenodo.21245733) (36). This is consistent with PI3K having access to nuclear substrates such as histone H2A for phosphorylation. Accordingly, we hypothesized that PI3K might moonlight as a checkpoint kinase in *Plasmodium*.

*Pf*PI3K is the only phosphoinositide 3-kinase of the six phosphoinositide kinases in *Plasmodium* (34): it is a well-validated, essential lipid kinase (37), with roles in nutrient uptake and vesicle trafficking (38, 39). However, its moonlighting as a protein kinase would not be unprecedented because other PI3Ks can act on proteins as well as lipids. For example, humans encode a large family of PI3Ks, and their type-1 PI3Ks can phosphorylate protein substrates like cytokine receptors, as well as auto-phosphorylating themselves (40, 41). The sole *Plasmodium* enzyme is a type-3 PI3K, and thus far, these are proven only to phosphorylate lipids (37).

**Fig 3:**
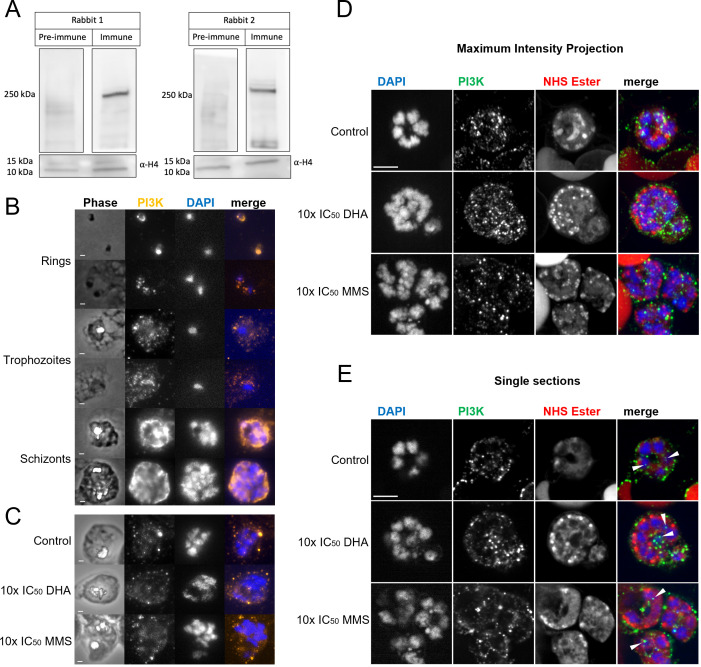
*Pf*PI3K is dispersed throughout the cell and does not visibly relocate to the nucleus upon DNA damage. (**A**) Western blots showing detection of *Pf*PI3K by two antisera raised to *Pf*PI3K peptides. Replicate lanes from the same blot of protein lysate from late-stage *P. falciparum* were exposed to either pre-immune or immune serum. Histone H4 was used as a loading control. (**B**) Immunofluorescence assay (IFA) showing location of *Pf*PI3K (detected by antiserum 1) in *P. falciparum*. Two representative cells are shown per stage. Scale bar, 1 μm. (**C**) IFA showing *Pf*PI3K in *P. falciparum* after DNA damage: 1 h treatment with 5× or 10× IC_50_ of DHA or MMS on late-stage parasites. One representative cell per condition is shown. Scale bar 1 μm. (**D**) Confocal microscopy showing *Pf*PI3K after expansion: maximum projections of representative cells in each condition, as in (**C**). DAPI, blue; *Pf*PI3K, green; NHS ester, red; scale bar, 10 μm. (**E**) Single sections from the confocal microscopy in (**D**): nuclear *Pf*PI3K signals are highlighted with arrows.

We sought orthogonal validation of the spatial proteomics via immunofluorescence after raising peptide antibodies to *Pf*PI3K (Fig. 3A; see Fig. S4B and C at https://doi.org/10.5281/zenodo.21245733). These antibodies specifically detected a protein of the correct >250 kDa size in western blot (Fig. 3A), while in immunofluorescence, they detected a protein in puncta throughout the cytoplasm, consistent with previous work that used an independently raised anti-PfPI3K antibody and detected similar puncta (39) (Fig. 3B). This highly dispersed location could obscure any minor nuclear signal, and we did not detect substantial re-location to nuclei upon DNA damage (Fig. 3C). We then used expansion microscopy to better resolve the location of *Pf*PI3K (Fig. 3D and E; see Fig. S5 at https://doi.org/10.5281/zenodo.21245733), observing that puncta grew more abundant as cells matured, and were dense in an area free of nuclei that is probably the food vacuole—a known hub for vesicle trafficking (Fig. S5). Importantly, some *Pf*PI3K was also detected inside nuclei in all cells imaged (Fig. 3E). Of note, we recently showed that the ATM and ATR homologs in *Toxoplasma* are likewise dispersed throughout the cell and do not substantially relocate to the nucleus after DNA damage, but they are nevertheless able to mediate checkpoint phenotypes such as histone phosphorylation (42).

We attempted to use the same antibodies to immunoprecipitate native *Pf*PI3K with any potential protein substrates, but only a single *Pf*PI3K peptide was detected across several attempts. It is probably highly unstable *in vitro*, or lacking suitable tryptic digest sites, because it was likewise completely undetected, despite its very large size, in two dedicated proteomic studies of the nuclear versus cytoplasmic proteomes in *P. falciparum* (43, 44), and it was only sparsely detected in spatial proteomics (36). Further validation of an intra-nuclear role for *Pf*PI3K would probably require methods focused less on the kinase itself and more on its targets—such as BioID for proximity-labeling of protein partners (45).

### The mediator of DNA-damage checkpoint responses in *Plasmodium* is sensitive to PIKK inhibitors

Assuming that a divergent PIKK-like kinase does exist in *P. falciparum*, we attempted to inhibit it with PIKK inhibitors. We chose two compounds with validated specificity for human ATM and ATR respectively (but not for human PI3Ks), KU-55933 and VE-821 (46, 47). If an ATM-like or ATR-like activity existed in *P. falciparum*—either in *Pf*PI3K or elsewhere—these compounds might specifically inhibit it.

**Fig 4:**
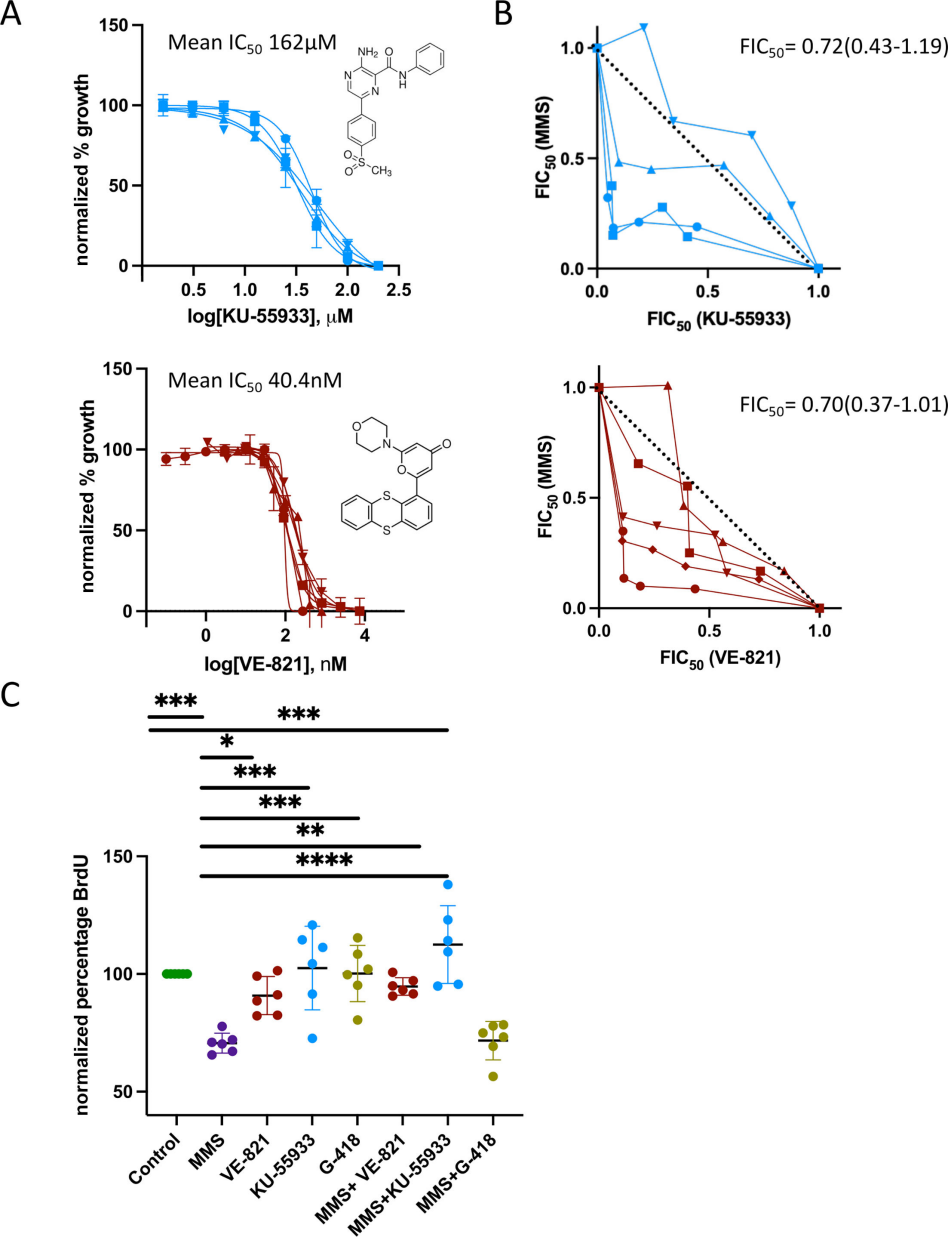
Inhibitors of human PIKKs synergize with the DNA-damaging agent MMS in *P. falciparum.* (**A**) Inhibition of parasite growth by VE-821 and KU-55933 (molecular structures of each inhibitor are shown). Growth curves from five independent malaria SYBR green I-based fluorescence (MSF) assays: percentage parasite growth is plotted as a function of drug concentration, and error bars are standard errors of the means. Mean IC_50_s from all experiments are shown (individual IC_50_ values from replicate experiments are in Fig. S1B at https://doi.org/10.5281/zenodo.21245733). (**B**) Isobolograms plotted as fractional inhibitory concentrations (FIC) of combinations of MMS and VE-821 or KU-55933, as determined by MSF assay. FIC calculations were completed for each combination by dividing the measured “apparent” IC_50_ values for individual drugs in the different combinations by IC_50_ values obtained when the drugs were used alone. Each curve represents a different biological replicate experiment. The mean FIC [ΣFIC(FIC_A_ + FIC_B_)] is shown, with range. The type of relationship was defined as moderately synergistic based on well-established criteria: ∑FIC < 0.5, substantial synergism; ∑FIC < 1, moderate synergism; ∑FIC ≥ 1 and <2, additive interaction; ∑FIC ≥ 2 and <4, slight antagonism and ∑FIC > 4, marked antagonism (48). (**C**) BrdU incorporation into 30–32 hpi trophozoites after treatment with MMS, VE-821, KU-55933, geneticin, and combinations of these, measured by ELISA. Parasites were treated with 10× IC_50_ of drug for 1 h, and BrdU was added for the latter 30 min. Six biological replicates are plotted, each completed in technical triplicate. Mean and standard deviation error bars are plotted, values normalized to control (set to 100%) in each replicate. Concentrations of drugs used: MMS 960 µM, VE-821 1.62 µM, KU-55933 400 µM, geneticin 1,500 µg/mL. Ordinary one-way ANOVA and post hoc Tukey’s multiple comparisons test of control vs all and MMS vs all was completed; *P*-values: *, <0.05; **, <0.01; ***, <0.001; ****, <0.0001; comparisons not shown, ns.

We first determined their 48 h IC_50_ values on *P. falciparum* (3D7 strain): KU-55933 had an IC_50_ in the micromolar range and VE-821 in the nanomolar range (Fig. 4Asee Fig. S1B at https://doi.org/10.5281/zenodo.21245733). Both inhibitors clearly synergized with MMS in a 48 h isobologram analysis, particularly strongly in the case of VE-821, suggesting activity in the same pathway (Fig. 4B). Control compounds that kill parasites but are not known to cause DNA damage—the antibiotics blasticidin and geneticin—did not synergize with MMS (see Fig. S6 at https://doi.org/10.5281/zenodo.21245733). Furthermore, when parasites were exposed to high-dose MMS (10× IC_50_, as in Fig. 1) simultaneously with KU-55933 or VE-821, the inhibitors ablated the acute reduction in DNA replication caused by MMS, whereas co-exposure to the control antibiotic geneticin did not (Fig. 4C). In this short 1 h assay, we used the PIKK inhibitors at only their 48 h IC_50_ levels, to minimize any negative effects from the inhibitors alone. Indeed, within the timeframe of the assay the inhibitors alone did not significantly affect DNA replication (Fig. 4C). Overall, these data strongly supported the existence of a cryptic PIKK activity, responsible for a genuine intra-S-phase checkpoint via in *trans* kinase activity.

### The antimalarial drug dihydroartemisinin induces DNA replication arrest in *P. falciparum*

**Fig 5:**
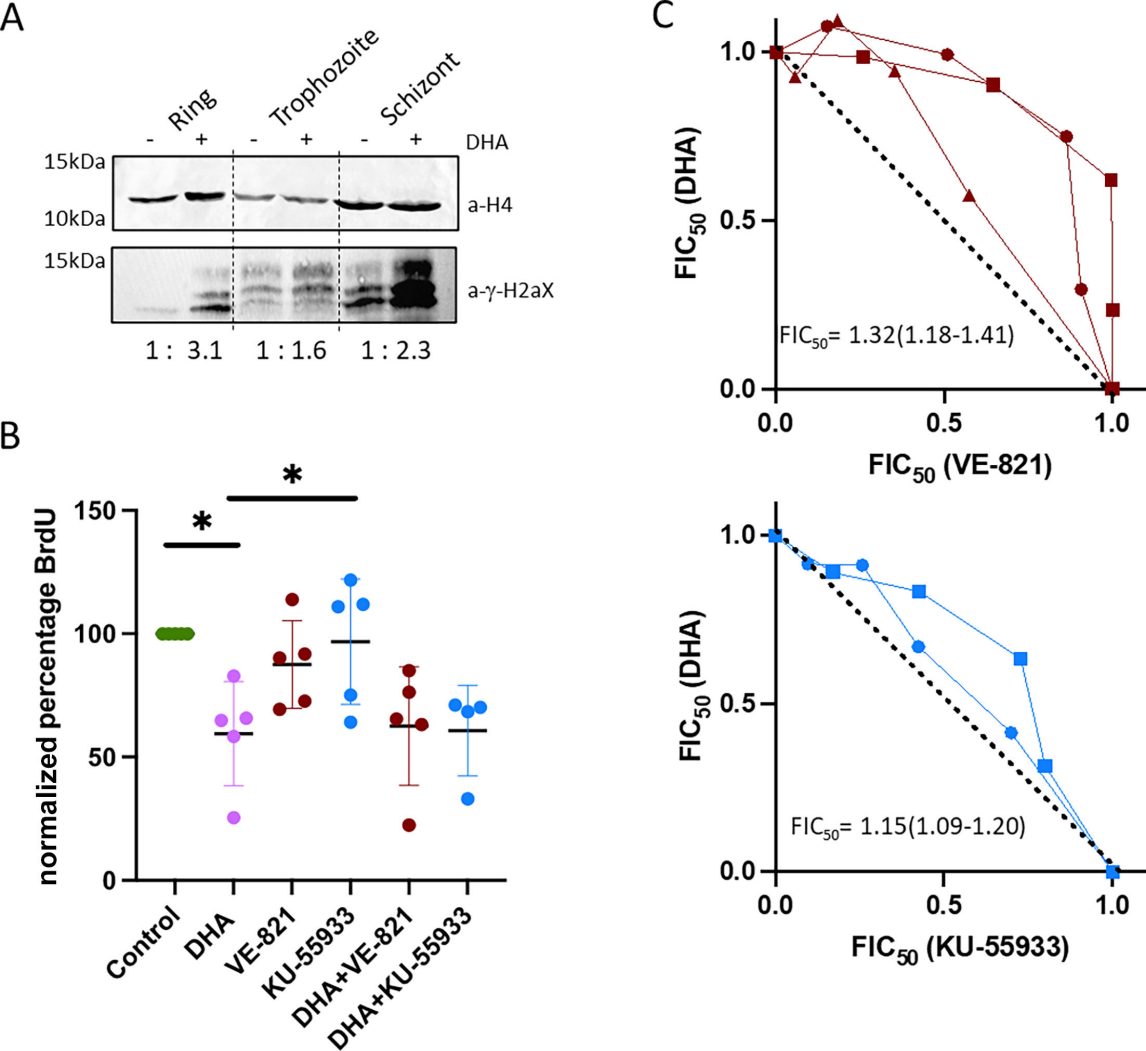
The antimalarial drug dihydroartemisinin induces a DNA replication arrest in *P. falciparum.* (**A**) Western blot of phosphorylated histones in ring, trophozoite, and schizont-stage parasites treated with DHA (4 h, IC_50_ [3 nM]). Phosphorylated histone signal was quantified vs control histone H4: relative quantification is shown below the blot. (**B**) BrdU incorporation into 30–32 hpi trophozoites after treatment with DHA, VE-821, KU-55933, and combination treatments. Parasites were treated with 10× IC_50_ of drug for 1 h, and BrdU was added for the latter 30 min. Five biological replicates were plotted, each completed in technical triplicate. Mean and standard deviation error bars are plotted; values were normalized to control (set to 100%) in each replicate. Concentrations of drugs used: DHA 45 nM, VE-821 1.62 µM, KU-55933 400 µM. Statistical testing by ordinary one-way ANOVA and post hoc Tukey’s multiple comparisons test of control vs all and DHA vs all; *P*-values: *, <0.05; comparisons not shown, ns. (**C**) Isobolograms plotted as Fig. 4B. Each curve is a biological replicate, and the mean FIC [ΣFIC(FIC_A_ + FIC_B_)] is shown, with range. The relationship between DHA and either VE-821 or KU55933 was defined as additive.

MMS is a useful, well-characterized DNA-damaging agent, but it is not an antimalarial drug. We were particularly interested in the potential DNA-damaging activity of antimalarial drugs, because if parasites do make checkpoint responses to such drugs, then their antimalarial activity might be potentiated by inhibiting checkpoint kinases. Artemisinin is such a drug: it has non-specific alkylating activity that may affect both proteins and DNA (49, 50). There are similarities between the parasite’s response to dihydroartemisinin (DHA, the active metabolite of artemisinin) and MMS in terms of DNA breakage (49, 51), delay in the parasite’s transcriptional cascade, and upregulation of DNA repair genes (21). Indeed, we found that DHA induced H2A phosphorylation, at all stages of the cycle but particularly in pre-replicative ring stages (~3-fold upregulation after 4 h exposure to IC_50_ DHA [Fig. 5A]). This confirmed that DHA does, indeed, cause damage that activates a putative histone kinase and, furthermore, that it can cause this damage prior to S-phase.

Using the replication-reduction assay, we saw that in replicating parasites, DHA caused an acute reduction in nascent DNA replication similar to MMS (Fig. 5B). However, unlike MMS, this was not ablated by the PIKK inhibitors KU-55933 and VE-821. Consistent with this difference, the inhibitors did not synergize with DHA in isobologram analysis (Fig. 5C). This suggested that although both DHA and MMS may damage DNA, they differ in either the type of damage or the cellular response thereto in terms of inducing an intra-S-phase checkpoint.

### Ring-stage survival after artemisinin damage is affected by PIKK inhibitors

Cell cycle checkpoint activity in *P. falciparum* may not be limited to an intra-S-phase checkpoint: most eukaryotic cells also operate a G1/S checkpoint, delaying S-phase entry if cells are damaged, starved, or stressed. This may be analogous to the “TGA” arrest or “dormancy” that occurs in ring-stage parasites after artemisinin treatment (14). We, therefore, set out to establish whether ring-stage dormancy induced by DHA actually represents a classical, G1/S checkpoint, responsive to the DNA damage caused in ring stages by DHA (as shown in Fig. 5A), and mediated by a potential PIKK-like kinase.

We conducted the ring-stage survival assay (RSA) on parasites treated with DHA in the presence or absence of PIKK inhibitors. In this assay, pre-replicative early-ring-stage parasites are damaged with a pulse of DHA: most of them die, but some successfully arrest and thence survive. The more parasites are able to do this, the higher the subsequent recovery of viable parasites. We reasoned that if successful arrest is due to PIKK-mediated checkpoint activity, then PIKK inhibitors should reduce ring-stage survival. These experiments were conducted in the 3D7 strain and also in the Cambodian strain MRA-1252, derived from a naturally arising DHA-resistant strain reverted back to a sensitive phenotype by specific correction of the resistance mutation in the *Kelch13* gene (52): its genetic background is otherwise that of a S.E. Asian DHA-resistant line.

**Fig 6:**
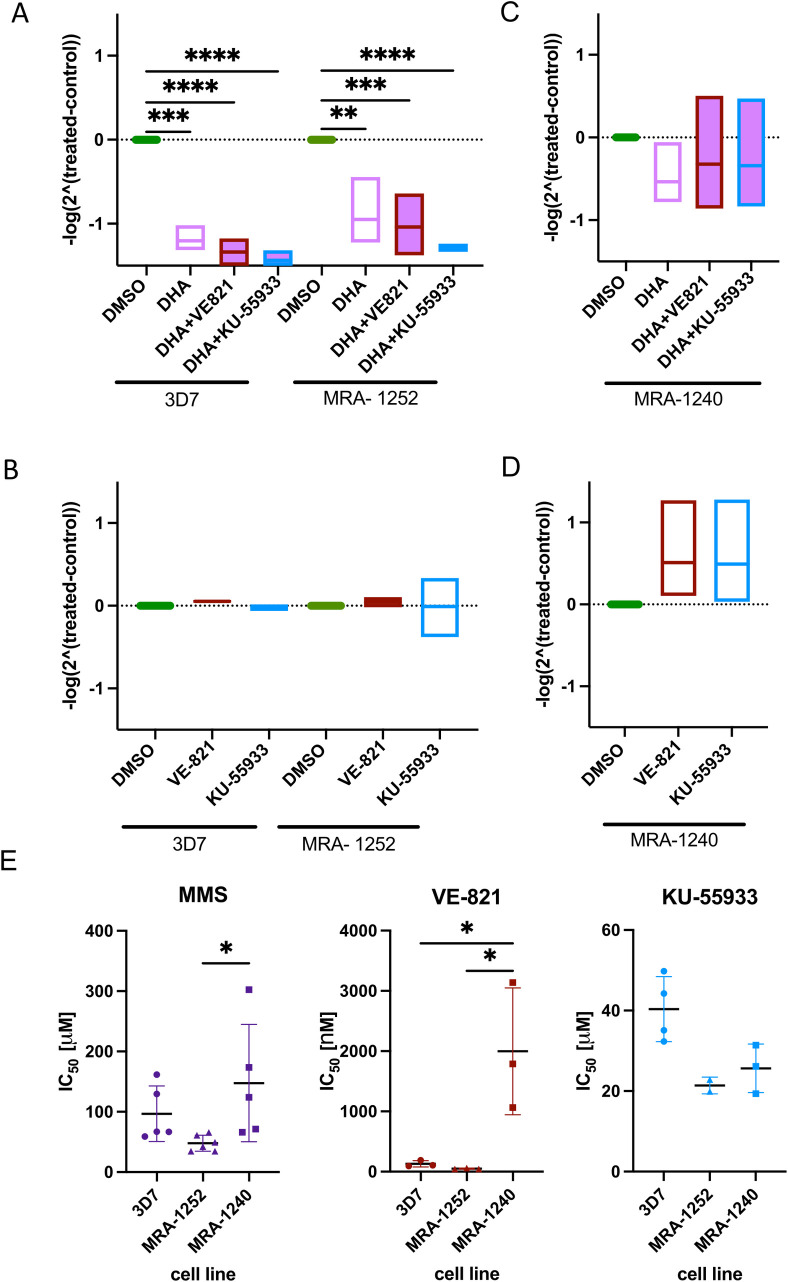
Ring-stage survival is affected by PIKK inhibitors. Data in panels** A–D** are from eRRSA assays on the strains 3D7, MRA-1252, and MRA-1240. Samples were treated with DMSO (the control condition), VE-821, KU-55933, DHA, and combinations of these. Drug concentrations: VE-821, 81 nM (0.5× IC_50_); KU-55933, 20 μM (0.5× IC_50_); DMSO, 0.02%; DHA, 700 nM. Values plotted are the means of −log(fold change = 2^DHA − DMSO^) for each treatment. The midline represents the mean and boxes show min to max from three biological replicates, each conducted in technical triplicate. Statistical testing was by ordinary one-way ANOVA and Dunnett’s multiple comparisons test, comparing all means to the mean value from DMSO- or DHA-treated samples. *P*-values: *, <0.05; **, <0.01; ***, <0.001; ****, <0.0001. Differences between DHA-treated samples ± PIKK inhibitors did not reach statistical significance, but the *P*-values for DHA + VE-821 or DHA + KU-55933 vs control were <0.0001, rather than 0.001 for DHA-alone versus control. (**A**) Ring-stage DHA-sensitive parasite strains, 3D7 or MRA-1252, were treated with DMSO (control condition), DHA, and inhibitors VE-821 or KU-55933 in combination with DHA for a 6 h pulse and then left to recover for 72 h. (**B**) Control experiments without DHA: ring-stage 3D7 or MRA-1252 parasites were treated with DMSO or inhibitors VE-821 or KU-55933 for a 6 h pulse and then left to recover for 72 h. (**C**) Ring-stages of the DHA-resistant parasite strain MRA-1240 were treated with DMSO (control condition), DHA and inhibitors VE-821 or KU-55933 in combination with DHA for a 6 h pulse, and then left to recover for 72 h. (**D**) Control experiment without DHA: ring-stage MRA-1240 parasites were treated with DMSO or inhibitors VE-821 or KU-55933 for a 6 h pulse and then left to recover for 72 h. (**E**) Inhibition of growth over a full 48 h growth cycle by MMS, VE-821, and KU55933 was measured in the strains 3D7, MRA-1252, and MRA-1240 via MSF assay. IC_50_s were calculated from at least three independent experiments, and all values are plotted, with the midline showing the mean and error bars showing standard deviation. MSF growth curves are in Fig. S1C at https://doi.org/10.5281/zenodo.21245733.

Both 3D7 and MRA-1252 are highly sensitive to artemisinin, so we used the “extended recovery ring-stage survival assay” (eRRSA), which detects recovered parasites very sensitively after 72 h via quantitative PCR (53). Fig. 6A shows that PIKK inhibitors, particularly KU-55933, did exacerbate the killing of ring-stage parasites by DHA. Differences were subtle and parallel assays reading the RSA with the less sensitive flow cytometric assessment of live/dead cells likewise showed marginal differences (see Fig. S7 at https://doi.org/10.5281/zenodo.21245733). However, in triplicate eRRSAs on two independent lines, 3D7 and MRA-1252, survival trended consistently downward when DHA was combined with either inhibitor—so the effect, although not strong, was highly reproducible. As above, we minimized any negative effects of the inhibitors themselves by using them at a low level: only half of the 48 h IC_50_. We confirmed that this exposure alone for 6 h did not affect parasite survival after 72 h (Fig. 6B).

These RSAs did not show conclusively that “TGA” after artemisinin damage represents a true G1/S checkpoint, but they did suggest that such a checkpoint, mediated by a cryptic PIKK, may contribute to ring-stage survival. Hence, the cryptic PIKK must be active throughout the cell cycle, not only in actively replicating trophozoites.

When the same assay was conducted on DHA-resistant parasites (the parent strain of MRA-1252, called MRA-1240), the result differed. As expected, DHA survival was much better in MRA-1240, and PIKK inhibitors had no negative effect on this survival (Fig. 6C). There was even a trend towards better growth and DHA survival when MRA-1240 was exposed to PIKK inhibitors (Fig. 6D). Hence, when a Kelch13 mutation was present, conferring DHA resistance, any contribution of checkpoint activity to parasite survival was apparently ablated.

### Artemisinin-resistant parasites are cross-resistant to MMS and VE-821, but not to KU-55933

We hypothesized that artemisinin-resistant parasites like MRA-1240 might not require “G1/S” checkpoint activity to survive DHA damage because they are more efficient at DNA repair. Several mutations in DNA repair genes have been identified in resistant strains (50) although the pathway(s) leading to improved DNA repair is not fully characterized. We checked whether the DHA-resistant line MRA-1240 was cross-resistant to MMS, and its IC_50_ was, indeed, significantly higher than that of the matched sensitive line MRA-1252 (and also higher than that of 3D7) (Fig. 6E, left panel; see Fig. S1C at https://doi.org/10.5281/zenodo.21245733). Since the point mutation in Kelch13 is the only difference between the two Cambodian MRA lines, a genetic background of enhanced DNA repair cannot fully account for this.

Another explanation could be that MRA-1240 has hyper-active checkpoint activity due to Kelch13-associated over-expression of PI3K (35) and that this stimulates hyperactive DNA repair. This explanation would be consistent with the Kelch13 mutant and revertant having different sensitivities to MMS. If so, the Kelch13 mutant state would also be expected to correlate with reduced sensitivity to PIKK inhibitors. Indeed, MRA-1240 was much less sensitive to the VE-821 inhibitor (Fig. 6E, middle panel; see Fig. S1C at https://doi.org/10.5281/zenodo.21245733)—so much so that it was difficult to measure a consistent IC_50_ via MSF assay. Curiously, however, the same line was not less sensitive to KU-55933 (Fig. 6E, right panel; Fig. S1C). This was the first instance in which VE-821 and KU-55933 had shown a clear difference in their effects on *P. falciparum*. It may suggest that in the MRA-1240/1252 genetic background (perhaps unlike 3D7), more than one checkpoint protein is required, and that one of the inhibitors has a broader inhibitory spectrum than the other. For example, *Pf*PI3K may, indeed, moonlight as a checkpoint kinase, uniquely unregulated after Kelch13 mutation and uniquely inhibited by VE-821, whereas *Pf*PI4K and/or other “off-target” kinases may also be needed when broader-spectrum damage induces more complex checkpoint responses, and KU-55933 may inhibit this broader kinase spectrum.

Validating these speculations would require challenging experiments, such as biochemical assays on recombinant PI3/4K proteins (which are ~250 kDa in size), or robust conditional manipulation of the two large, essential genes encoding *PfPI3K* and *PfPI4K* in parallel. We did make several attempts to tag the *PfPI3K* gene for conditional knockdown, but the enzyme’s active site is encoded at the extreme 3′ end and may become mis-folded after 3′ tagging, because all our attempts failed. Similar efforts by other groups to tag or manipulate this gene were likewise unsuccessful or gave no phenotype (54, 55), while a recently published mutant that irreversibly deletes the 3′ end of the *PfPI3K* gene was lethal (56). The very large size of the gene (6.6 kb) also precludes its overexpression or dominant-negative overexpression, as well as recombinant protein production for enzyme-activity assays. Overall, these large and essential genes proved near-intractable to molecular genetics. Thus, their putative PIKK-like activity was not conclusively evidenced or disproved, but the dual nuclear/cytoplasmic location of *Pf*PI3K does suggest that it could moonlight on nuclear substrates.

## DISCUSSION

This work provides the first evidence that *Plasmodium* parasites during blood-stage schizogony can enforce an intra-S-phase checkpoint. The checkpoint is evidenced by an acute reduction in the rate of DNA replication and increased phosphorylation of histone H2A in a position analogous to the canonical DNA damage marker H2AX. *P. falciparum* was previously shown to slow its cell cycle progression after DNA damage (21), but it was not clear if this occurred through cell cycle checkpoint activity (partly because the *Plasmodium* system lacked validated DNA damage markers, signals, or transducers thereof). Markers still remain limited—there are, for example, no cyclin markers for cell cycle phases, and the H2A phosphorylation that was reported as a DNA damage marker in ring stages (27) is, in our hands, a consistent but low-dynamic-range marker in trophozoite stages, probably because background levels of H2A-P are higher in actively replicating parasites. However, the ability to measure nascent DNA replication in *Plasmodium* (57) does now allow intra-S-phase checkpoint activity to be measured. Thus, we showed that DNA replication was acutely arrested by two distinct alkylating agents, MMS and the antimalarial drug DHA.

The transducer(s) of checkpoint activity in *Plasmodium* remains cryptic. *Plasmodium* spp. lack identifiable orthologs of PIKK kinases in contrast to many apicomplexans including *T. gondii* (42). Nevertheless, such activity is apparently present because the reduction in replication caused by MMS was ablated by well-validated inhibitors of human PIKKs. Hence, it must be induced in *trans* by a kinase-mediated signaling cascade (also consistent with H2A phosphorylation), rather than occurring simply because alkylated DNA bases physically block the replisome. Interestingly, DHA differed from MMS in this respect: it also induced H2A phosphorylation, and it also affected DNA replication, but PIKK inhibitors did not significantly ameliorate this. Possibly, the acute effect of DHA—which is a much larger molecule than MMS*—*is primarily to block replisomes via bulky DNA adducts (a documented effect of alkylating agents in human cells [19]). If such adducts cannot be quickly bypassed whether a checkpoint kinase is active or not, then PIKK inhibitors might not ameliorate the acute reduction in DNA replication.

Other, more complex, explanations are also possible for the difference in response to MMS vs DHA. For example, slow DNA replication can, in general, stimulate compensatory activation of extra replication origins (58), but when damage to DNA is sensed, checkpoint kinases act in *trans* to suppress such origin firing (19). If checkpoint kinases are inhibited, then extra origins may fire regardless, hence MMS-damaged cells may keep replicating and encountering more damage, so genome replication ultimately fails to complete. This would explain how MMS and PIKK inhibitors ultimately synergize in cell killing. Perhaps, the broader-spectrum damage that DHA causes to proteins as well as DNA (49), and/or the oxidative stress that it generates (51) means that compensatory origin firing does not happen, whether a checkpoint is activated or not. Hence, PIKK inhibitors would not synergize with DHA in cell killing. To evidence these rather complex hypotheses, precision measurement of DNA replication dynamics at single-molecule resolution after different types of damage will be needed. This has been achieved in human cells (19) and is now in development for *Plasmodium*.

Turning to the possibility of a pre-replicative G1/S checkpoint, the evidence for this being mediated by a PIKK-like kinase is tantalizing albeit not conclusive. In the RSA, which measures DHA killing of pre-replicative rings, PIKK inhibitors exacerbated parasite killing. The effect was modest, so if a classical (kinase-enforced) G1/S checkpoint does exist, it is probably not the sole defining factor in survival of artemisinin-induced dormancy. Nevertheless, the trend was consistent in triplicate assays across two genetically distinct DHA-sensitive strains, 3D7 and MRA-1252. Checkpoint kinase activity could, therefore, help to protect ring-stage cells, perhaps by detecting DNA damage, stimulating repair, and preventing S-phase entry until repair is complete. By contrast, a DHA-resistant strain showed no effect of PIKK inhibitors. This may be because it can already suppress DHA activation in early rings and, thus, protect itself from DNA damage, or alternatively, it may still sustain some damage, but be rescued by hyperactive DNA repair (50). Consistent with this latter hypothesis, it was recently shown that when *Pf*PI3K is inducibly knocked out in a DHA-resistant Kelch-13 mutant background, the parasites become less DHA resistant—perhaps because they can no longer resist or repair DHA-related DNA damage when the mediating kinase (*Pf*PI3K) is lost (56).

If there is, indeed, hyperactive DNA repair in the MRA-1240 line, it cannot be conferred *per se* by a DHA-resistant genetic background because the matched strain MRA-1252 was not cross-resistant to MMS, whereas MRA-1240 was. However, it could be because the Kelch13 mutation, uniquely present in MRA-1240, confers hyper-checkpoint activity, thus stimulating hyperactive DNA repair. In any case, these data raise the intriguing possibility that artemisinin drugs could synergize with checkpoint inhibitors in killing ring-stage parasites; the caveat being that this might only work well in sensitive, not resistant, strains.

It is an attractive possibility that *Pf*PI3K is the cryptic checkpoint kinase, particularly if Kelch13 mutant strains do contain more *Pf*PI3K (35). However, we were not yet able to prove or disprove this, owing to the extreme difficulty of manipulating the large, essential *PfPI3K* gene. Broad-spectrum inhibitors such as wortmannin were used in previous work (39), but these are slow-killing and unable to separate lipid-kinase from putative protein-kinase activity. We used PIKK inhibitors instead, hoping to isolate a putative protein kinase activity. The fact that our PIKK inhibitors did not synergize with DHA in MSF assays, whereas specific inhibitors of distinct human PI3Ks (i.e., lipid kinases) have been reported to do so (59), may suggest that this succeeded and that VE-821 and KU-55933 do target a distinct activity in *Plasmodium*. Of course, they may affect irrelevant off-targets, i.e., multiple or unexpected kinases, or even non-kinases. As an example, a repurposed human-kinase inhibitor was recently deployed to target the PK6 kinase in *Plasmodium*, but it actually targeted haem detoxification as well (60). More likely, however, is that these inhibitors genuinely inhibit a *Plasmodium* checkpoint kinase—which may reside in *Pf*PI3K, *Pf*PI4K, or another radically divergent protein. *Plasmodium* contains many kinases whose roles are yet-unknown (61), and the *Plasmodium* cell cycle is driven by markedly divergent kinases such as CRK4 (62), so it is entirely possible that *Plasmodium* checkpoints are enforced by a novel kinase(s) as well.

In conclusion, we present new evidence for measurable cell cycle checkpoint activity in *Plasmodium*. We also raise the considerable challenge of unambiguously identifying its mediators in this divergent eukaryotic parasite.

## MATERIALS AND METHODS

### Parasite cultures

*Plasmodium falciparum* cultures were grown in 2% or 4% hematocrit human erythrocytes (Research Red Cells obtained from the NHS Blood & Transplant service), in RPMI 1640 (Sigma, R4130) supplemented with 2.3 g/L sodium bicarbonate, 50 mg/L hypoxanthine (Sigma, H9377), 25 μg/L gentamicin (Melford Laboratories, G38000-1), 2.5 g/L Albumax II, and 5% human serum. Parasites were cultured under a 1% O_2_/3% CO_2_/96% N_2_ gaseous atmosphere at 37°C.

### Parasite strains

Parasite strains used were 3D7, 3D7 pTK, MRA-1240, and MRA-1252. 3D7 pTK carries a thymidine kinase transgene to allow incorporation of bromodeoxyuridine (BrdU) for monitoring DNA replication (57). MRA-1240 and MRA-1252 are matched artemisinin-resistant and sensitive Cambodian field lines, respectively (52), obtained through BEI Resources (NIAID, NIH). Parasites were synchronized as in reference 63.

### Database sampling and phylogenetic analysis

The phylogeny (Fig. 2) for ATM, ATR, TOR, PI3K, and PI4K homologs was inferred by following a computational workflow (https://github.com/camwallerlab/Methods-for-phylogenetic-analysis-of-plastid-translocons) developed as part of a previous study (64). In summary, this workflow involved sampling a custom protein sequence database that included data for major eukaryotic groups from UniProt (65) and the Marine Microbial Eukaryote Transcriptome Sequencing Project (66), as well as data for apicomplexans and close relatives from VEuPathDB (67) and two previous studies (68, 69). This custom database was then sampled for ATM, ATR, and TOR homologs using the program blastp (BLAST +version 2.11.0 [70]), for which characterized homologs of these proteins from selected model organisms were used as search queries. The data set of possible ATM, ATR, and TOR homologs obtained was then clustered with CD-HIT (71) to remove highly similar sequences, thereby reducing overall redundancy of the data set. PI3K and PI4K homologs from *Plasmodium* and other apicomplexans, as well as characterized homologs from *Saccharomyces cerevisiae* and *Homo sapiens,* were incorporated into the data set as a phylogenetic outgroup for the ATM, ATR, and TOR proteins. Iterative rounds of alignment were then performed using mafft (MAFFT version 7.475 [72]), conserved site selection using trimal with “gappyout” mode (trimAl version 1.4 [73]), and tree inference with FastTreeMP (FastTree version 2.1.11 [74]) using the default settings. For each iteration, sequences that aligned poorly or were so dissimilar from the rest of the sequences in the data set that they were unlikely to be ATM, ATR, or TOR homologs, but rather false positives of the described sampling strategy were manually identified and removed from the data set. The final curated data set that these iterations obtained was then aligned using mafft-linsi (MAFFT version 7.475 [72]), and conserved sites were selected using trimal with “gappyout” mode (trimAl version 1.4 [73]). A phylogeny was then inferred from this data set using the program iqtree2 (IQ-TREE version 2.1.2 [75]) with 1,000 ultrafast bootstrap replicates (UFBoot2 [76]) and using the best-fitting model, LG + F + I + G4 (77) that was chosen according to the Bayesian Information Criterion by ModelFinder (78), all implemented within iqtree2. The protein sequences, alignment, and tree inference output files used to generate the phylogeny are provided as source data.

### BrdU enzyme-linked immunosorbent assay

ELISA was carried out as per reference 57. In brief, tightly synchronized *P. falciparum* 3D7 pTK parasites were collected at 4% parasitemia and 4% hematocrit at 28 hpi, 32 hpi, and 36 hpi. They were labeled with BrdU (100 μM, Sigma) for 30 min and then collected for ELISA, or treated with MMS at 10× IC_50_ (960 μM) or 20× IC_50_ (1,920 μM) for 30 min prior to BrdU labeling. In further experiments, parasites at 30–32 hpi were exposed to either MMS alone (10× IC_50_), MMS + VE-821 (1× IC_50_), MMS + KU-55933 (1× IC_50_), VE-821 alone, KU-55933 alone, DHA alone (10× IC_50_), DHA + VE-821, DHA +KU-55933 or no drugs, then processed as above.

### Immunofluorescence

Air-dried thick blood smears were fixed with 4% paraformaldehyde in PBS for 10 min and permeabilized for 10 min with 0.05% Triton in PBS. Slides were then blocked for 1 h in 1% BSA/PBS. The primary antibody (1:200 immune or preimmune antiserum) was added in blocking buffer for 1 h under a coverslip. After 3 × 5 min washes in PBS, the secondary antibody was added to slides for 1 h. The slides were washed with PBS for 5 min, 120 μL of 2 μg/mL DAPI solution was added for 10 min, and then the slides washed again with PBS for 5 min. Coverslips were mounted on slides in Prolong Diamond (Invitrogen) and allowed to dry overnight at 4°C. Images were acquired using a Nikon Eclipse Ti widefield microscope with a Nikon objective lens (Plan APO, 100×/1.45 oil), and a Hamamatsu C11440, ORCA Flash 4.0 camera. Images were processed using the NIS-Elements software and ImageJ (v.1.51w).

### Immunofluorescence with expansion microscopy

Expansion microscopy was performed according to Liffner et al. (5).

Twelve millimeter round coverslips (Fisher, Cat# NC1129240) were treated with poly-d-lysine (1 h, 37°C), washed twice with MilliQ water, and placed in the wells of a 12-well plate. One milliliter of parasite culture was added to the well containing the coverslip for 15 min at 37°C. Culture supernatants were removed, and parasites were fixed with 1 mL of 4% (vol/vol) paraformaldehyde in PBS for 15 min at 37°C. Following fixation, coverslips were washed three times at 37°C with PBS before being treated with 1 mL of 1.4% (vol/vol) formaldehyde/2% (vol/vol) acrylamide (FA/AA) in PBS. Samples were then incubated at 37°C overnight.

Monomer solution (19% [wt/wt] sodium acrylate [Sigma, Cat# 408220], 10% [vol/vol] acrylamide [Sigma, Cat# A4058, St. Louis, MO], 0.1% [vol/vol] N,N′-methylenebisacrylamide [Sigma, Cat# M1533] in PBS) was typically made the night before gelation and stored at −20°C overnight. Prior to gelation, FA/AA solution was removed from coverslips, which were washed once in PBS. For gelation, 5 µL of 10% (vol/vol) tetraethylenediamine (TEMED; Thermo Fisher, Cat# 17919) and 5 µL of 10% (wt/vol) ammonium persulfate (APS; Thermo Fisher, Cat# 17874) were added to 90 µL of monomer solution and briefly vortexed, and then 35 µL was pipetted onto parafilm and coverslips were placed (cell side down) on top. Gels were incubated at 37°C for 30 min before transfer to wells of a 6-well plate containing denaturation buffer (200 mM sodium dodecyl sulfate [SDS], 200 mM NaCl, 50 mM Tris, pH 9). Gels were incubated in denaturation buffer with shaking for 15 min, before separated gels were transferred to 1.5 mL tubes containing denaturation buffer. The tubes were incubated at 95°C for 90 min. Following denaturation, gels were transferred to 10 cm petri dishes containing 25 mL of MilliQ water for the first round of expansion and placed onto a shaker for 2 × 15 min, changing water in between. Gels were subsequently shrunk with 2 × 15 min washes in 25 mL of 1× PBS, before being transferred to 6-well plates for 30 min of blocking in 3% BSA-PBS at room temperature.

After blocking, gels were incubated with primary antibodies (1:200 immune antiserum) and diluted in 3% BSA-PBS, overnight. Gels were washed three times in PBS for 10 min before incubation with secondary antibodies (1:1,000) and DAPI diluted in 3% BSA-PBS for 3 h. Gels were again washed three times in PBS, incubated in PBS with NHS Ester (4:1,000) for 1 h and washed three times in PBS for 10 min, before being transferred back to 10 cm Petri dishes for re-expansion with three 30 min MilliQ water incubations.

Gels were either imaged immediately following re-expansion or stored in MilliQ water until imaging, using a Nikon Eclipse Ti2 widefield microscope with a Nikon objective lens (Plan APO, 60×/1.40 oil), 405 and 488 lasers, and a Hamamatsu C11440, ORCA Flash 4.0 camera. Confocal images were taken using an Evident IXplore IX83 SpinSR spinning disk confocal microscope with Olympus objective (60×/1.5NA, UPLAPO60XOHR, 0.11 mm, oil), 405, 488, and 594 lasers and 2 Hamamatsu Fusion BT sCMOS cameras. Z-stacks were taken with a depth range of 2–20 µm, at interval range of 0.2–0.6µm (depending on the parasite stage). Images were processed using the NIS-Elements software, cellSens software, and ImageJ (v.1.51w).

### Malaria SYBR Green-1 fluorescence assay

MSF assays were conducted as in reference 79. One hundred micromolar chloroquine (Merck) was used as a control for parasite death. Data were analyzed and graphs plotted in GraphPad Prism.

### Isobolograms

Parasites were prepared as for MSF assays. Isobologram design followed the procedure in reference 80. Serial dilutions were planned so that the IC_50_s of each drug would be in the second twofold dilution out of 6 total dilutions. IC_50_s for each combination solution were calculated using sigmoidal best-fit curves assigned in Graphpad Prism. Fractional inhibitory concentrations (FIC values) were calculated in Excel (81) and isobolograms plotted in Graphpad Prism. Source data for the isobolograms are provided in Fig. S8 at https://doi.org/10.5281/zenodo.21245733.

### Ring-stage survival assay

Parasite cultures at 8%–10% schizonts were synchronized using percoll and Compound 2 as in reference 63 and then allowed to reinvade. Early ring-stage (0–3 hpi) parasites at 0.5% parasitemia, 2% hematocrit were exposed for 6 h to 700 nM DHA or 0.02% DMSO (vehicle control) under normal conditions, before washing three times with incomplete media, resuspending in complete media, and incubating under the same conditions for a further 66 h. In addition to the DHA 700 nM pulse, VE-821 and KU-55933 inhibitors were used at 0.5× IC_50_ with DHA, or by themselves, throughout the 6 h pulse period. Seventy-two hours after initial drug exposure, 20 μL was frozen for RT-PCR as per eRRSA protocol (53).

### qPCR-based RSA analysis

qPCR amplification was used to quantify live parasites in the eRRSA (53). The Phusion Blood Direct PCR kit was used (ThermoFisher, cat # F547L) and supplemented with 1× SYBR Green I (ThermoFisher) and 50 nM Low ROX (ThermoFisher) according to the manufacturer’s instructions. qPCR amplification was measured using the fast mode of the ABI 7900HT, with a 20 s denaturation at 95°C, followed by 35 cycles of 95°C for 1 s, 62.3°C for 30 s, and 65°C for 15 s. Cycle threshold (Ct) values were calculated; then the fold change was calculated by determining the mean difference in Ct (ΔCt) for the three technical replicates between the untreated and DHA-treated samples by applying the following equation: fold change = 2^ΔCt of treated – ΔCt of untreated^. This was −log transformed, and data were plotted in GraphPad Prism.

### Flow cytometry-based RSA analysis

Flow cytometry wash buffer “FC wash” (2% FBS [Gibco] in HBSS [calcium, magnesium, no phenol red, Gibco]) was prepared. The remaining 180 μL of each RSA assay, not harvested for eRRSA, was centrifuged, and packed cells were washed three times in 200 µL of FC wash and then resuspended in FC wash to make a 10% hematocrit suspension. Twenty-five microliters of suspension from each assay (conducted in technical triplicate) was mixed with 25 µL of 0.6× SYBR Green I (SYBR) with 1.5 nM MitoTracker Deep Red FM (MT, Invitrogen) in FC wash. Compensation controls were prepared with suspension from one DMSO-treated triplicate, labeled with either 0.6× SYBR, 1.5 nM MT, or FC wash alone. Samples were incubated in the dark at 37°C for 30 min, washed three times in 200 µL, and resuspended in 180 µL of FC wash.

Ten thousand events of SYBR positive cells were captured at a flow rate of 25 µL/min on an Attune NxT Acoustic Focusing Cytometer (Invitrogen). SYBR and MT fluorescence were detected on the BL1 (488 nm excitation, 530/30 nm emission, 340 V) and RL1 (638 nm excitation, 670/14 nm emission, 510 V) channels, respectively. Debris and doublets were excluded by gating on a forward scatter area (FSC-A) vs side scatter height (SSC-H) plot and forward scatter area (FSC-A) vs forward scatter height (FSC-H). SYBR/MT quadrant plots were generated using the Attune NxT software v4.2.0 (Invitrogen).

### Antibody production

Peptides were designed from *P. falciparum* PI3K (PF3D7_0515300) with help from Eurogentec using parameters such as size, amino acid composition, hydrophobicity, and secondary structure. Two peptides were chosen—one predicted to be in the catalytic domain and one upstream of this domain, both predicted to be surface-displayed. N-terminal cysteines were added to the peptide sequences to target the coupling site at the carrier protein.

Antibodies were raised by Eurogentec. Two rabbits were bled for pre-immune sera, inoculated with both peptides, and then further immunized with three booster inoculations at 12 days, ~1 month, and ~2 months. Immune serum was collected after ~9 weeks.

### Western blotting

Parasites were extracted from infected erythrocytes by mixing with 0.5 volume of 0.2% saponin in PBS and incubating for 10 min on ice. Saponin lysates were centrifuged at 15,000 *× g* at 4°C for 10 min, washed 3× in ice-cold PBS, centrifuged at 15,000 *× g* for 5 min, and resuspended in PBS before freezing at −20°C. Parasites obtained by saponin lysis were resuspended in 1× RIPA buffer (150 mM sodium chloride, 1.0% NP-40 or Triton X-100, 0.5% sodium deoxycholate, 0.1% SDS, and 50 mM Tris, pH 8) with protease inhibitors (ROCHE, Complete Mini) added directly before use and then freeze-thawed three times. Cell debris was removed by centrifugation at 4°C, 13,000 *× g*, and supernatant was mixed with 1× NuPage LDS Sample Buffer (ThermoFisher: NP0007), supplemented with 0.1% 2-mercaptoethanol, and then boiled at 90°C for 5 min. Protein lysates were resolved on 4%–15% Tris-glycine gels (Bio-Rad Mini—Protean TGX) for 1–2 h at 100 V in 1× TGS buffer. Wet transfer was completed onto nitrocellulose membrane 0.2-μm-pore-size (Amersham Protran, GE Healthcare) at 60 V for 45 min.

Membranes were blocked with 3% BSA TBST for 1 h, washed once in TBST, and incubated overnight in primary antibody diluted in blocking buffer at 4°C. Blots were washed 3 × 5 min in TBST and incubated in a secondary antibody diluted 1:5,000 in blocking buffer for 1 h. Blots were washed again (3 × 5 min, TBST), developed using SuperSignal West Pico Plus Chemiluminescent Substrate, and imaged on a gel imager (Azure, Q500). Some membranes were stripped prior to re-blocking (10 mL 20% SDS and 12.5 mL 0.5M Tris HCl, pH 6.8, added to 77.5 mL distilled water, with 0.8 mL β-mercaptoethanol added just before use). Five milliliters of stripping buffer (warmed to 50°C) was added to a blot and incubated at 50°C for 45 min with agitation. The stripping buffer was removed, and the membrane was washed under a running tap for 1–2 min and then washed 3 × 5 min with TBST. The membrane was then ready for re-blocking.

Antibodies used were pre-immune or immune sera for *Pf*PI3K peptides, diluted 1:200, with secondary goat anti-rabbit HRP 1:5,000 (Abcam, ab97080); or rabbit α-γ-H2aX (Cell signaling, 9718S) with goat α-rabbit IgG (H + L) cross-adsorbed secondary antibody, Alexa Fluor 594 (Invitrogen, A-11012).
